# Regulation on Dual Interfaces of QD with ETL and HTL by Guanidine‐Based Ligands Enable High‐Performance Blue Quantum Dot Light‐Emitting Diodes with 24.3% External Quantum Efficiency

**DOI:** 10.1002/advs.202512478

**Published:** 2025-09-25

**Authors:** Yanfang Ren, Yunqi Wang, Yan Fang, Xiaohong Jiang, Ke Cheng, Zuliang Du

**Affiliations:** ^1^ National and Local Joint Engineering Research Center for High‐efficiency Display and Lighting Technology Key Lab for Special Functional Materials of Ministry of Education and School of Nanoscience and Materials Engineering Henan University Kaifeng 475004 China

**Keywords:** charge balance, dual interfaces modification, hole injection, passivating interfacial defects, quantum dot light‐emitting diodes (QD‐LEDs)

## Abstract

The poor efficiency and stability of blue quantum dot light‐emitting diodes (QLED) hinder its practical applications in full‐color displays. Insufficient hole injection and excessive surface defects in quantum dots (QD) layer remain the primary challenges limiting the performance of blue devices. Herein, a dual interface modification strategy is proposed to enhance the performance of blue QLED by synergistically regulating both the electronic transport layer (ETL)/QD and hole transport layer (HTL) HTL/QD interfaces. At the HTL/QD interface, the introduction of guanidine sulfamate (GAS) ligands passivates QD surface defects while reducing the hole injection barrier, thereby improving hole injection efficiency in the low‐bias region. Meanwhile, at the QD/ETL interface, Guanidine chloride (GACl) ligands are incorporated to passivate interfacial defects, suppress leakage current, and suppress excessive electron injection, thus enhancing hole transport efficiency within the QDs layer. The synergistic effect of bilateral GA‐based ligands can simultaneously enhance the hole injection efficiency based upon improving the hole transport efficiency, significantly increasing the radiative recombination ratio during device operation. As a result, the dual‐ligand modified blue QLEDs achieve a remarkable improvement in external quantum efficiency (EQE) from 16.6% to 24.3%, and a sevenfold enhancement in operational lifetime.

## Graphical abstract

Insufficient hole injection in the quantum dot emission layer is the primary challenge limiting the performance of blue devices. Herein, a bilateral ligands modification strategy is proposed to enhance the hole injection efficiency on the basis of improving the hole transport efficiency, effectively improves the carrier recombination efficiency, with the EQE increasing from 16.5% to 24.3%.

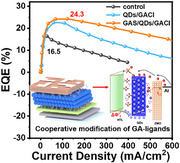

## 1 Introduction

Quantum dot light‐emitting diodes (QLEDs) are expected to become the next‐generation mainstream lighting and display technology due to the advantages such as high color purity, high efficiency, and low cost.^[^ ^1^, ^2^, ^3^ ^]^ After years of research and development, the current external quantum efficiency (EQE) for red, green, and blue QLEDs has reached 38.2%,^[^ ^4^ ^]^ 28.8%,^[^ ^5^ ^]^ and 24%,^[^ ^6^ ^]^ respectively. T_95_@1000 cd m^−2^ operation lifetime (the time for the luminance to decrease to half of the initial luminance) for red, green, and blue QLEDs stands at 24 100 h,^[^ ^4^ ^]^ 17 700,^[^ ^7^ ^]^ and 227 h,^[^ ^8^ ^]^ respectively. Obviously, the EQE and stability of blue‐emitting QLEDs considerably lag significantly behind the red and green counterparts. Further improvement in the EQE and stability of blue QLEDs is earnestly needed to realize large‐scale industrial applications in the field of display.^[^ ^9^, ^10^, ^11^, ^12^, ^13^, ^14^ ^]^

Excessive electron injection, insufficient hole injection are the main primary challenges limiting the performance of blue QLEDs devices. Compared with red and green quantum dots (QDs), blue QDs have smaller sizes, leading to higher surface defect densities and deeper VB energy levels, which makes fluorescence quenching more severe at QDs emissive layer (EML) related interface and larger hole injection barrier at the interface between the hole transport layer (HTL) and QDs EML.^[^ ^8^, ^10^, ^15^, ^16^ ^]^ From the perspective of device control engineering, passivating surface defects, suppressing electron excessive injection, and enhancing hole injection are the main ways to improve device performance.^[^ ^17^, ^18^, ^19^, ^20^, ^21^, ^22^ ^]^ At the QDs /electronic transport layer (ETL) interface, Sargent et al.^[^ ^17^ ^]^ replaced long‐chain ligands with short‐chain Cl ion ligands through in situ immersion to passivate interface defects and improve charge interlayer transport efficiency. Du et al.^[^ ^23^ ^]^ utilized dipole aromatic amine functionalized molecules with different molecular polarities to reduce excessive electron injection. At the HTL/QDs interface, Wang et al.^[^ ^24^ ^]^ constructed a double‐ HTL structure to form a gradual energy level gradient, which can facilitate the hole injection, achieving balanced carriers. Bae et al.^[^ ^25^ ^]^ introduced molecular dipole ligands at the interface between the HTL and the QD EML, reducing the hole injection barrier by controlling the dipole direction. Maximizing the efficiency of hole injection and improving the properties of interface states through device control engineering remain highly challenging research tasks.

In our previously reported work, the ionic compound small molecule polydentate Guanidine chloride (GACl) ligand was introduced to regulate the properties of the QD/ETL interface, providing an additional extraction force for hole transport, which effectively increases the efficiency of hole transport within the QDs layer resulting in improvement both of efficiency and lifespan.^[^ ^26^ ^]^ Meanwhile, we found the different hole injection processes under high and low bias voltage: below flat‐band voltage (V_F_), hole injection is controlled by the interfacial barrier, primarily governed by tunneling and thermionic emission; above V_F_, the interfacial barrier is eliminated, and hole injection efficiency is determined by the transport efficiency within the QDs. Obviously, the existence of a hole injection barrier limits the improvement of hole injection efficiency.^[^ ^23^, ^27^ ^]^ From the perspective of overall device performance improvement of blue QLDs, increasing the hole injection efficiency while improving the transfer efficiency within the hole QDs layer is necessary and crucial.

Based on the findings and concise physical model of hole injection of our previous work, in this work, we propose a dual‐interface ligand modification strategy to further enhance the performance of blue QLEDs. At the QDs/ETL interface, GACl ligands are introduced to passivate interfacial defects, suppress excessive electron injection, and enhance hole transport efficiency within the QDs layer. Meanwhile, at the HTL/QDs interface, guanidine sulfamate ligands (GAS) are incorporated to reduce the hole injection barrier and simultaneously passivating interface defects. The synergistic effect of the dual‐interface GA‐based ligands simultaneously enhances the injection and transport efficiency of holes. As a consequence, the electrooptical conversion performance of blue QLEDs experienced substantial enhancement, with the EQE increasing from the initial 16.3% to a state‐of‐the‐art 24.3%. This improvement was accompanied by elevated luminance (44 100 cd m^−2^), enhanced current efficiency (21.4 cd A^−1^), and high device stability.

## 2 Results and Discussion

Blue CdZnSe/CdZnS/ZnS core‐shell QDs served as the light‐emitting materials in the construction of blue QLEDs. The absorption and photoluminescence (PL) spectra of the QDs solution are illustrated in Figure S1 (Supporting Information). The first exciton absorption peak, located at 455 nm, confirms the presence of strong quantum confinement. The Gaussian PL spectra of the QDs solution, centered at 468 nm, signify vibrant blue emission. The X‐ray diffraction (XRD) pattern indicates that the quantum dots used exhibit a face‐centered cubic sphalerite structure.^[^ ^16^, ^22^ ^]^ It can be seen from the TEM image that the size of the QDs used is uniform and well dispersed.

**Figure 1:**
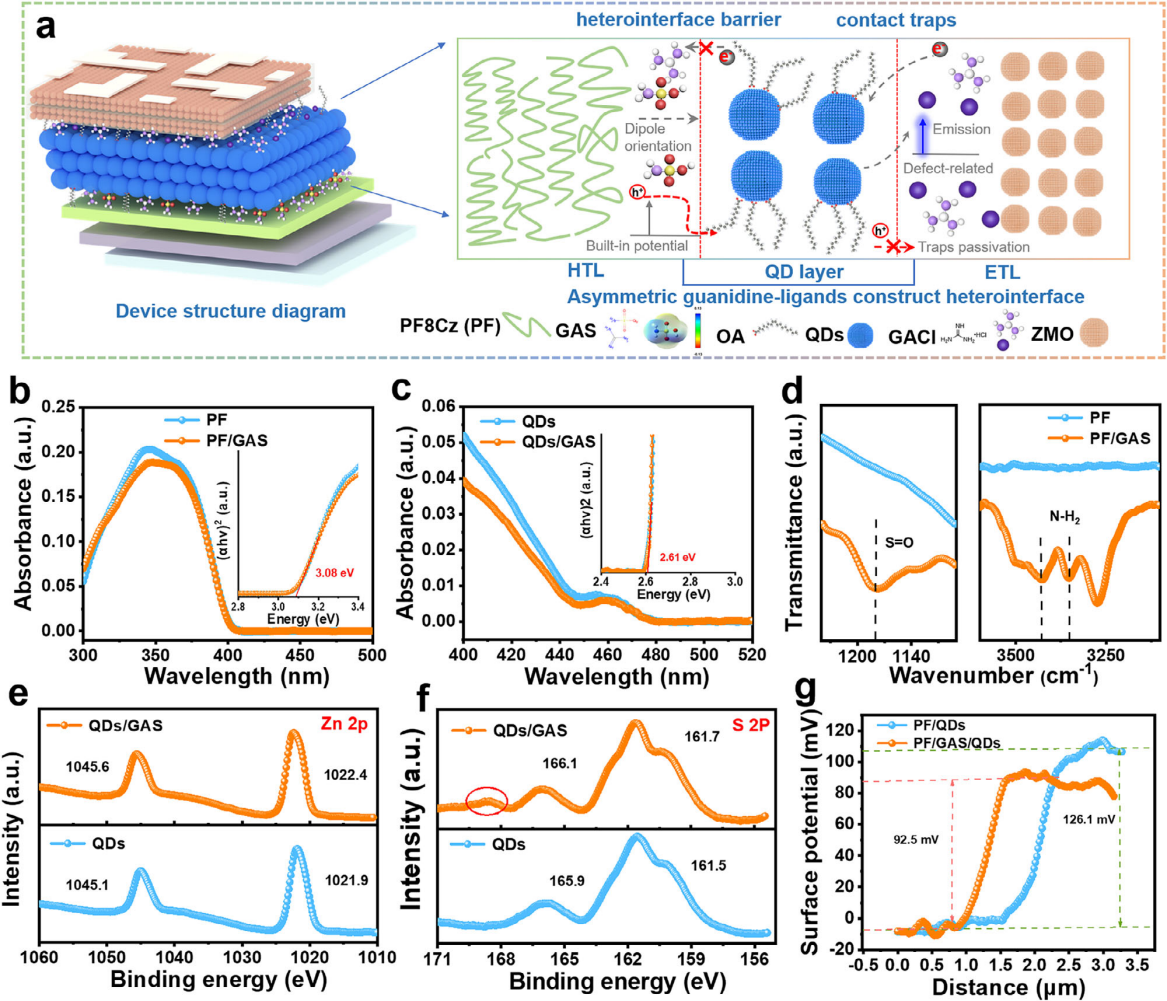
a) Schematic of stacked QLEDs with device architecture: ITO/PEDOT: PSS/PF/ QDs/ZMO/Al, and schematic cross‐sectional diagram of QLEDs based on QD film contact interface mediation. b) UV–vis absorption spectra of pristine PF film and GAS‐treated PF film. c) UV–vis absorption spectra of pristine QDs film and GAS‐treated QDs film. d) FTIR spectra of pristine PF film and GAS treated PF film. High‐resolution X‐ray photoelectron spectroscopy (XPS) results: e) the Zn 2p spectra and f) the S 2p spectra obtained for the pristine and GAS‐treated QDs film. g) Surface potential distribution curve of QDs and QD/GAS films, surface partially covered with PF8Cz HTL.

In the stacked structure of the device, the ETL/QD/HTL interface defects and intrinsic heterojunction barriers significantly influence the overall performance of blue QLEDs. As shown in **Figure** **1**a, the bipolar GAS molecule is introduced for interface modification at the HTL/QD interface. GAS solution is spin‐coated onto the top surface of the PF8Cz (PF) film. Subsequently, the QDs EML was spin‐coated in situ, allowing the GAS ligand to access the interface between the PF HTL and QDs EML. The GAS is an amphoteric ionic small molecule, consisting of a positively charged part composed of three amino (─NH_3_) branches, and a negatively charged part including a sulfonic acid (─SO_3_) anchoring group at the head and a simple hydroxyl (─OH) group at the tail. The strong coordination ability of the ─SO_3_ group can form a stable coordination with the Zn^2+^ on the surface of the QDs, effectively reducing interface defect states. At the ETL/QD interface, we continued to introduce the GACl ligand to passivate interface defects and modulate carrier injection, based on previous work.^[^ ^26^ ^]^

From the AFM images (Figure S2, Supporting Information), the average roughness of the PF8Cz (PF) film is 0.41 nm. After inserting the GAS ligand layer, the average roughness of the PF8Cz/GAS composite film is reduced to 0.34 nm, with a slight increase in the film thickness. Compared to the control QD film, the roughness of the PF8Cz/GAS/QD composite film is almost identical, indicating that the introduction of the GAS ligand does not disrupt the flatness of the HTL, thereby not affecting the subsequent QD film deposition. Microregion currents of QDs films, measured by conductive Atomic Force Microscopy (c‐AFM), showed that the conductivity of the film increases slightly after GACl and GAS treatment.

To explore the influence of GAS ligands on the interface properties of PF/QDs, the characteristics of the functional layer before and after GAS modification were tested by UV–vis absorption spectra. After GAS treatment, the absorption spectra of the PF film (Figure 1b) and QD film (Figure 1c) show only slight changes, further proving that the GAS ligand does not damage the films. The inset shows the Tauc’ plot obtained from the UV–vis spectra, demonstrating that the introduction of the GAS ligand layer does not change the bandgap. As shown in the Fourier transform infrared spectroscopy (FTIR) (Figure 1d), after the PF film was treated with GAS, the peaks at 3349 and 3433 cm^−1^ corresponding to ─NH_2_ symmetric and asymmetric vibrational modes, respectively, indicating the existence of GAS.^[^ ^28^ ^]^ The peak at 1179 cm^−1^ corresponds to the S═O double bond in the sulfonic group, indicating that the GAS ligand has been deposited on the PF8Cz HTL layer.^[^ ^29^ ^]^

To clarify the effect of the GAS ligand on the QD film, we used XPS to characterize the QD/GAS composite film. After GAS treatment, the QD film displayed a distinct N 1s peak (Figure S3, Supporting Information), further confirming the presence of the GAS ligand. As shown in Figure 1e, after GAS treatment, the binding energy of Zn 2p 3/2 and 2p 1/2 increased by 0.5 eV, shifting from 1021.9 eV and 1045.1 eV to 1022.4 eV and 1045.6 eV, respectively. For the S 2p orbitals (Figure 1f), the binding energy increased by 0.3 eV, with peaks shifting from 161.5 eV and 165.9 eV to 161.7 eV and 166.1 eV, and a new peak appeared at 168.7 eV corresponding to the ─SO_3_ functional group.^[^ ^22^ ^]^ The results suggest that the organic cation guanidine (GA^+^) and the ─SO_3_ group in the GAS ligand strongly coordinate with the vacancy defects of the QD surface, effectively passivating the interface defects at the HTL/QD junction.^[^ ^30^ ^]^

To further investigate the impact of the GAS ligand on the interfacial barrier at the PF HTL/QD junction, the surface potential and valence band energy levels of the films are characterized using ultraviolet photoelectron spectroscopy (UPS) and scanning kelvin probe (SKP) measurements. Figure S4 (Supporting Information) shows the UPS spectra of the PF8Cz film before and after GAS deposition. The introduction of GAS has little effect on the valence band energy level of the PF8Cz HTL, with a slight downward shift of 0.1 eV in the valence band position, as determined by the formula (E_Φ_ = 21.2–[E_cut‐off_–E_F_]).^[^ ^25^ ^]^

The surface potential of the stacked films is further characterized by scanning kelvin probe microscopy (SKPM).^[^ ^31^ ^]^ The surface potential images of the QD films before and after GAS treatment are shown in Figure S5 (Supporting Information). The surface potential of the control QD film is ≈−480 mV, while the QD/GAS film has a surface potential of ≈−440 mV. The higher surface potential of the QD/GAS film, along with the smaller absolute value of its Fermi level, indicates that the introduction of the GAS ligand causes an upward shift in the energy levels of the QD film.^[^ ^32^ ^]^ Figure S6 (Supporting Information) shows the surface potential images of PF8Cz HTL partially covering QD and QD/GAS films. After the introduction of the GAS ligand, the interface potential difference decreases from 126.1 to 92.5 mV (Figure 1g). The binding energy and dipole moment of the ligand molecules at the HTL/QD interface reduce the interface potential difference, effectively lowering the hole injection barrier and promoting hole transport across the heterojunction interface.^[^ ^25^ ^]^

**Figure 2:**
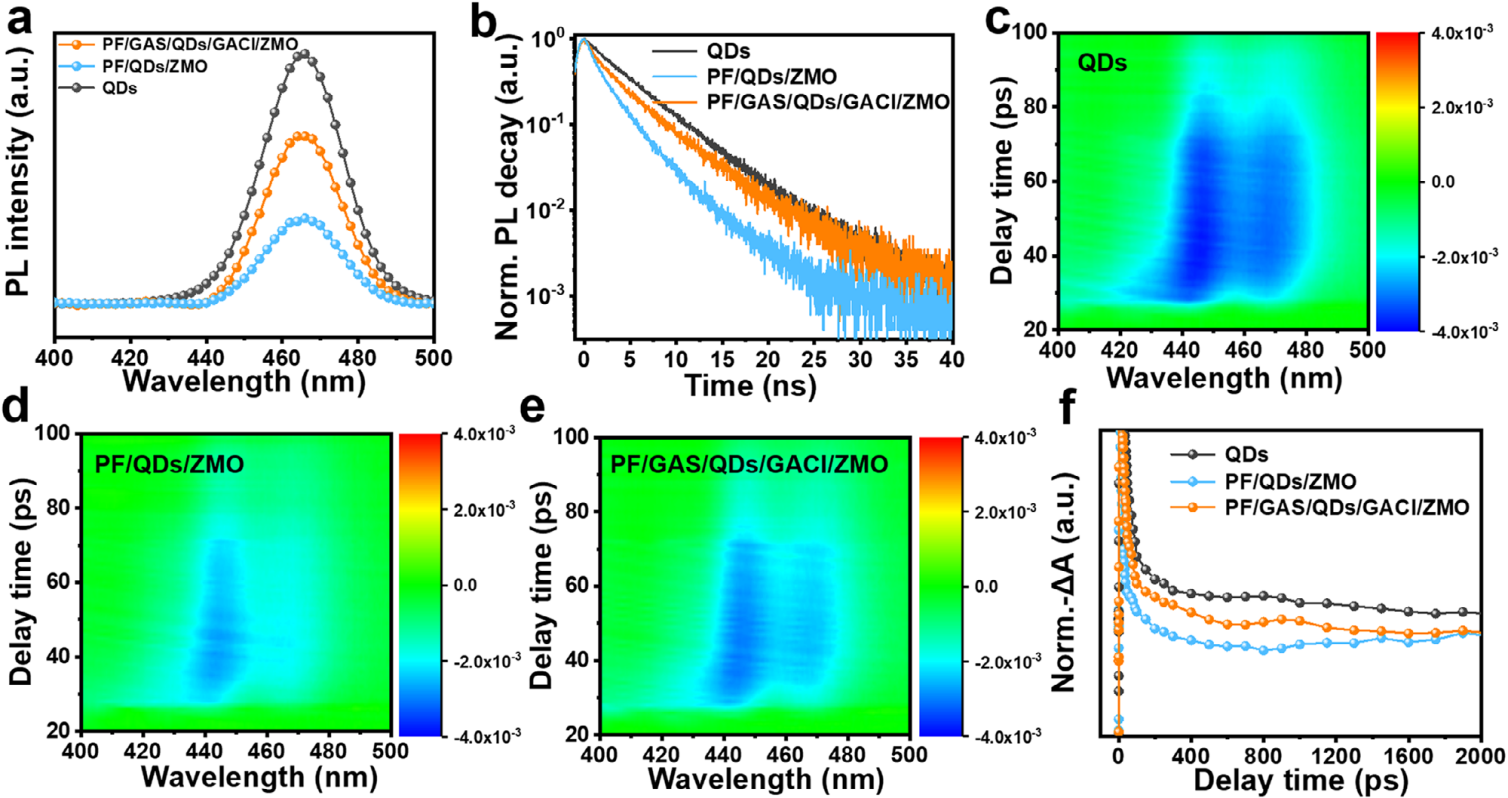
Optical properties of control QD film, PF/QD/ZMO composite film, and PF/GAS/QD/GACl/ZMO composite film. a) PL curves, b) TrPL curves, c) The 3D color plots of transient absorption (TA) spectra of QD film. d) The 3D color plots of TA spectra of PF/QD/ZMO composite film. e) The 3D color plots of TA spectra of PF/GAS/QD/GACl/ZMO. f) Comparison of TA bleach recovery kinetics.

To further evaluate the impact of bilateral ligands on the QD films, detailed optical characterization was performed, including photoluminescence (PL), time‐resolved photoluminescence (TrPL), and transient absorption (TA) spectroscopy. Figure S7a (Supporting Information) shows the PL spectra of the QD and PF/QD composite films. The PL intensity of the PF/QDs composite film is significantly lower than that of the original QD film, indicating that fluorescence quenching occurs at the HTL/QD interface (with emission peaks at 401 and 425 nm corresponding to PF8Cz HTL).^[^ ^33^ ^]^ After introducing the GAS ligand layer, the fluorescence intensity increases significantly. Similarly, Figure S7b (Supporting Information) shows the PL spectra of the QD and QD/ZMO composite films. The PL intensity of the QD/ZMO film is significantly lower than that of the original QD film, but the introduction of the GACl ligand partially suppresses the fluorescence quenching in the QD film.^[^ ^34^ ^]^ **Figure** **2**a shows the PL spectra of the QD film, PF/QD/ZMO film, and the PF/GAS/QD/GACl/ZMO composite film after the introduction of bilateral GA‐based ligands. The introduction of GAS and GACl ligands to the PF/QD and QD/ZMO interfaces results in a noticeable enhancement in the PL intensity. This improvement is due to the dual‐passivation effect of the bilateral GA‐based ligands, which passivate the surface/interface defects on both sides of the QD layer and reduce the interfacial fluorescence quenching. TrPL characterization further confirms the defect passivation effect of the bilateral ligands on the heterojunction interface. The TrPL curves for the QD, HTL/QD, and QD/ETL composite films are shown in Figure S7c (Supporting Information). When the charge transport layers on both sides are in direct contact with the QDs, the fluorescence lifetime of the QD film decreases significantly from 5.0 to 3.0 ns. This shortened fluorescence lifetime indicates the occurrence of exciton electron transfer at the QD/charge transport layer interfaces. After the introduction of bilateral GA‐based ligands at the heterojunction interfaces, the fluorescence lifetime of the QD film increases significantly to 4.6 ns, indicating that the dual‐side ligand treatment prevents the electron transfer to the charge transport layers, effectively suppressing non‐radiative recombination.

Femtosecond TA spectroscopy was used to study the transient energy level structure and relaxation dynamics of the QD films. The 3D TA spectra of the QD film are shown in Figure 2c, where a prominent negative probe bleaching peak is observed at 448 nm, corresponding to the steady‐state absorption peak, which originates from the ground‐state bleaching (GSB) of the excitonic states at the band edge.^[^ ^35^ ^]^ The 3D TA spectra of the PF/QD/ZMO composite film are shown in Figure 2d. Compared to the QD film, the transient absorption peak positions remain unchanged, but the corresponding intensity significantly decreases. After the introduction of the GA‐based bilateral ligands on both sides of the QDs, the 3D TA spectrum of the PF/GAS/QD/GACl/ZMO composite film shows an increase in intensity, indicating that the GA‐based ligands effectively passivate defects at both interfaces. To further verify the defect passivation effect of the dual‐side ligands, we analyzed the photogenerated exciton bleaching recovery dynamics, as shown in Figure 2f. Compared to the QD film, the PF/QD/ZMO composite film exhibits a significant reduction in photogenerated relaxation time at 448 nm, which is attributed to interface charge transfer. After the dual GA‐based ligand treatment, the PF/GAS/QD/GACl/ZMO composite film exhibits slower decay at this peak, indicating that the surface defect states that can capture photogenerated charge carriers within the bandgap are reduced. This demonstrates that the dual GA‐based ligands effectively suppress the charge transfer at both sides of the QD film, which is consistent with the TrPL results.

**Figure 3:**
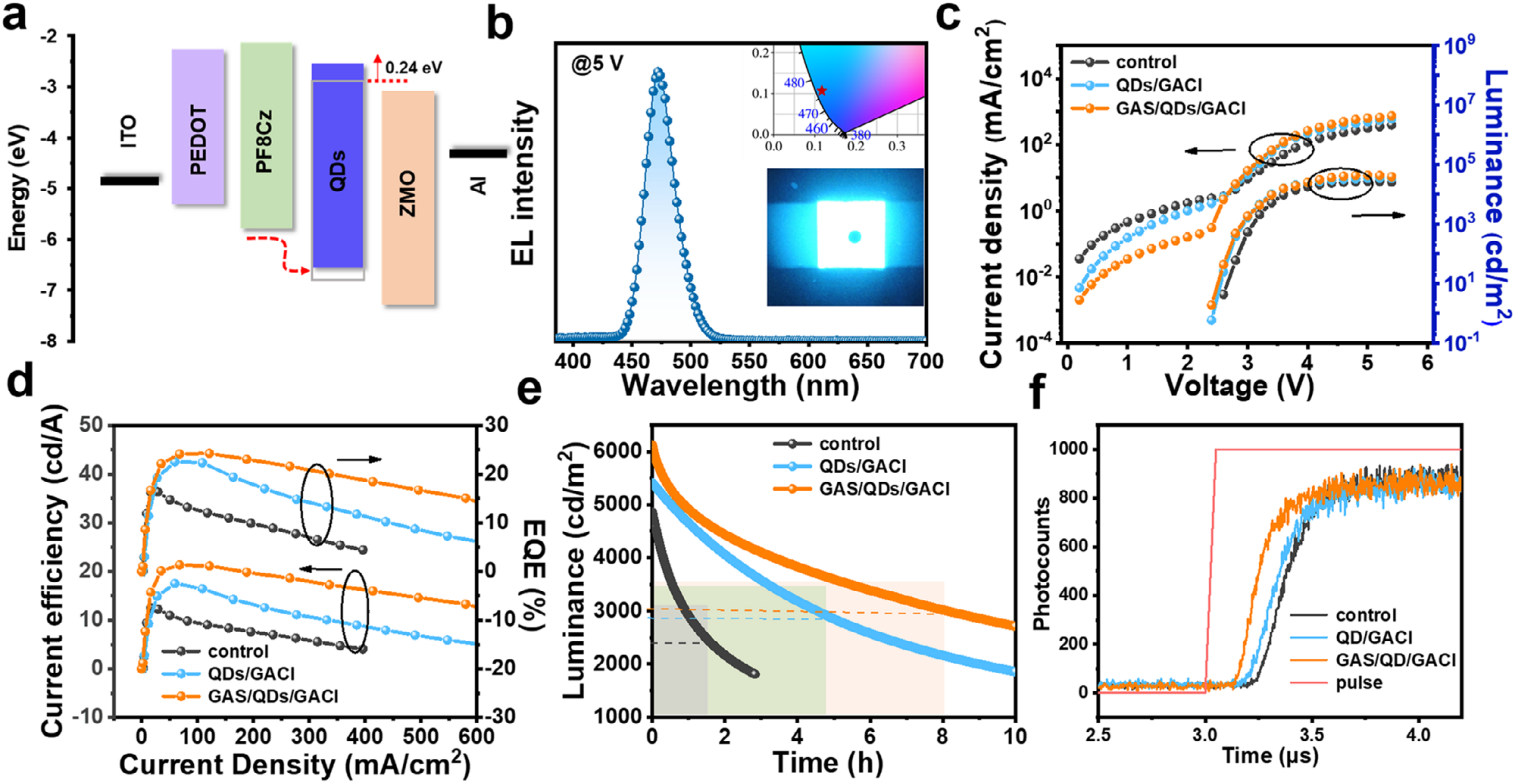
Performance of QLEDs. a) Energy‐band diagram of the QLEDs. The grey square indicates the energy level of the pristine QDs film. b) EL spectrum at applied voltage of 5 V, the inset was the corresponding CIE coordinates. c) Voltage‐dependent current density (left axis) and luminance (right axis). d) Luminance‐dependent current efficiency (left axis) and the EQE (right axis). e) Operational stability of the QLEDs under a constant current. f) Partial enlargement of the TrEL spectrums of different devices.

To further investigate the impact of bilateral GA‐based ligands on the electroluminescent (EL) performance of blue QLEDs, we fabricated devices using a fully solution‐processed method, with structures of ITO/PEDOT:PSS/PF8Cz/original QDs/ZMO/Al or ITO/PEDOT:PSS/PF8Cz/dual GA‐based ligand‐treated QDs/ZMO/Al. The energy level diagram of the functional layers in the QLEDs is shown in **Figure** **3**a. At the QD/ETL interface, after GACl treatment, the conduction band (CB) and valence band (VB) of the top QDs shift upward by 0.24 eV.^[^ ^26^ ^]^ The upshift of the CB increases the electron injection barrier, effectively blocking electron injection. At the HTL/QDs interface, the GAS ligand effectively reduces the interface barrier, promoting hole injection. Figure 3b presents the EL spectrum of the QLEDs under a 5 V bias, with an emission peak at 472 nm. The inset shows the corresponding CIE coordinates (0.12, 0.10), indicating pure blue emission.

Figure 3c shows the current density–voltage (J–V) and brightness–voltage (L–V) characteristics of the fabricated QLEDs. The J–V curve shows that, after GA‐based ligand modification at both the bottom and top of the QD emission layer, the leakage current is significantly reduced, and the turn‐on voltage is slightly lower. After reaching the turn‐on voltage, the current density and brightness increase rapidly. Compared to the control group, the QLED devices with dual ligand modification exhibit significant improvements in overall performance. The maximum luminance increased from 19740 to 44100 cd m^−2^, the current efficiency rose from 12.4 to 21.3 cd A^−1^, and the EQE improved from 16.6% to 24.3%. To verify the reproducibility of the dual‐ligand modification effect, we conducted a statistical analysis of the maximum EQE for 20 devices (Figure S9, Supporting Information), with an average value of 22.97%. The device lifetime under constant current conditions (with the current corresponding to the maximum EQE of the device) is tested to assess the improvement in the operational lifetime due to the dual‐ligand modification. As shown in Figure 3e, based on an acceleration factor of 1.64, the T_50_ lifetime of the QLEDs with bilateral dual‐ligand treatment increased from 933 to 6617 h under an initial brightness of 100 cd m^−2^. The performance parameters extracted from the devices are summarized in Table S2 (Supporting Information).

To further explore the charge injection, transport, and recombination mechanisms, we used the Transient Electroluminescence (TrEL) testing system to study the EL process in QLED devices. The TrEL system consists of a pulse signal generator, a digital oscilloscope, and a time‐correlated single‐photon counter (Figure S10a, Supporting Information). The square voltage signal used by the TrEL system has a frequency of 70 kHz, a pulse width of 5 µs, and a response time of 50 ns. Figure S10b (Supporting Information) shows the transient absorption spectrum of a conventional QLED device. The spectrum curve can typically be divided into four parts: i) Signal delay process, compared to the periodic pulse signal, the QLED devices EL emission signal has a certain delay time. This delay time, defined as τ_d_, is the time it takes for minority carriers (typically holes) to inject from the electrode into the QD emission layer. The delay is the point where the tangent of the rising edge of the EL emission signal intersects the tangent at zero time of the EL intensity; ii) EL emission signal rise, this is the process where the carrier concentration in the QD emission layer gradually increases; iii) EL steady‐state process, this is when the number of photons emitted by the device reaches its maximum value (saturation), and the carrier dynamics achieve a dynamic balance, iv) EL fast decay process, this occurs when the pulse voltage is turned off, and the remaining carriers in the device recombine. The TrEL spectra of QLED devices are tested before and after ligand modification under a voltage of 2.8 V. After bilateral ligand modification, a noticeable change was observed in region (i), the signal delay process. As shown in the magnified view of the QLED device in Figure 3f, the delay time τ_d_ was significantly shortened, indicating that the time for hole injection into the QD emission layer was reduced. This demonstrates that ligand modification can effectively regulate carrier injection.

**Figure 4:**
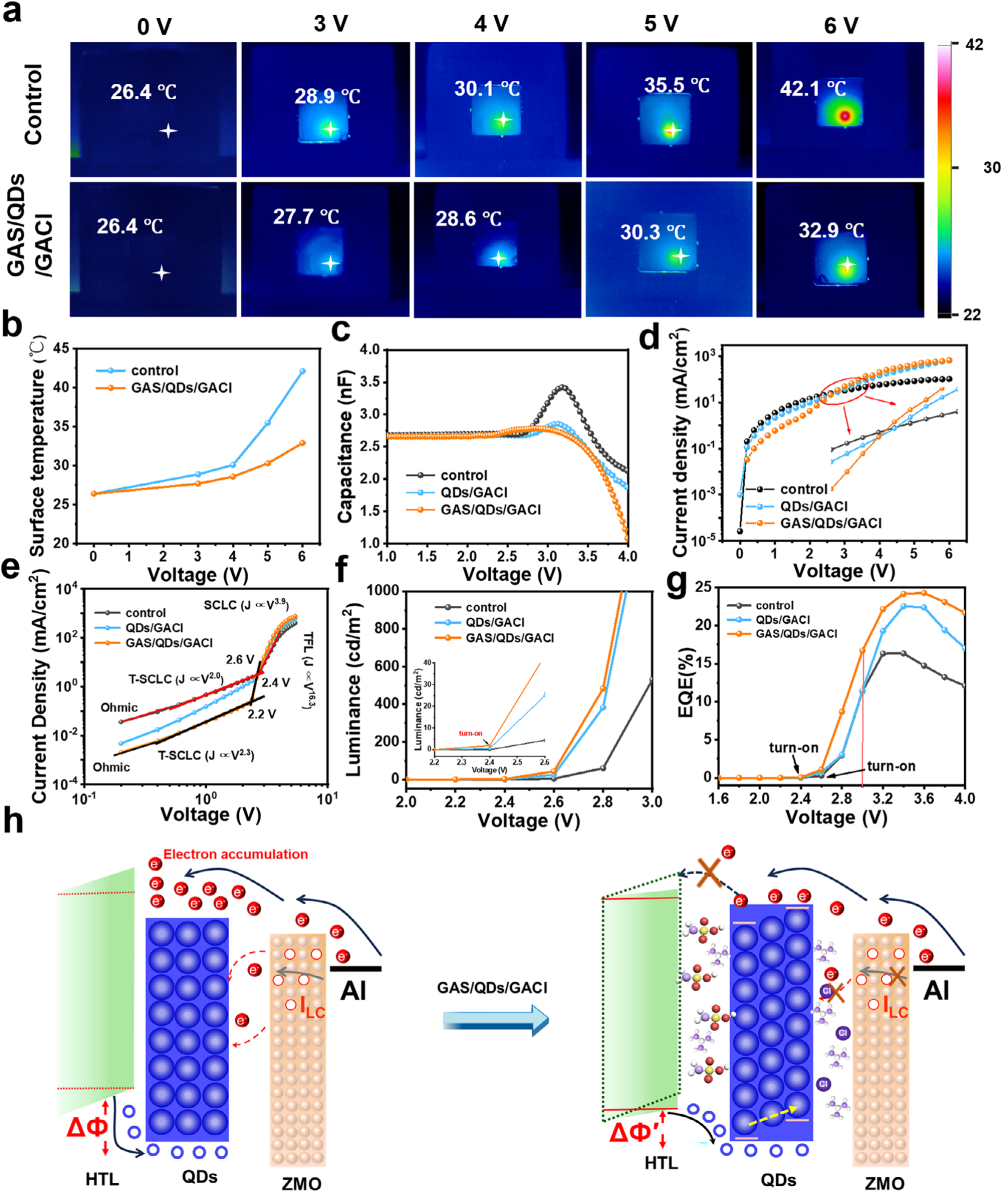
a) Surface temperature images of QLEDs based on bilateral GA‐ligand modification at various voltages. b) Surface temperature curves of QLEDs based on bilateral GA‐ligand modification at various voltages. c) C–V characteristics of QLEDs based on bilateral GA‐ligand modification. d) J–V characteristics of the HODs based on bilateral GA‐ligand modification. e) J–V curve of QLEDs. f) Enlarged view of the L–V curves. g) The EQE‐V curves. h) Schematic diagram of carrier transfer and recombination in QLEDs device.

**Figure** **4** shows the real‐time temperature images of the QLEDs device before and after bilateral Ga‐based ligand modification under different bias voltages, and records the increase in surface temperature of the device after 30 min of operation at room temperature (Figure 4b). The surface temperature of the control group QLEDs device increased by 15.7 °C during the process when the bias voltage increased from 0 to 6 V. For the QLED device modified with bilateral GA‐based ligands, the surface temperature only increased by 6.5 °C. The modification of Ga‐based ligands effectively inhibited the generation of Joule heat. (Note: Due to the relatively poor thermal conductivity of the functional layer materials, the internal temperature of the device is slightly higher than the measured temperature).

To further validate the carrier regulatory effect of bilateral Ga‐based ligands, the C–V characteristics are tested of the QLED devices during device operation.^[^ ^36^, ^37^ ^]^ Figure 4c shows the C–V characteristics of both the control group and the GA ligand‐treated QLED devices at a frequency of 10 kHz. The C–V characteristic curve of the control QLEDs device clearly reflects the charge injection and recombination process during the operation of the standard device. In the low‐voltage region, the hole injection is insufficient, and the carrier recombination efficiency is low. The obvious electron accumulation occurs inside the device, which affects the improvement of device performance. The hole injection increases as the voltage continues to rise. The improvement of carrier recombination efficiency leads to the consumption of accumulated electrons, and the device capacitance decreases accordingly. After the treatment with the bilateral ligand Ga‐based ligands, the peak capacitance decreased, indicating an increase in the hole injection efficiency and alleviating the accumulation of internal charges in the device. This is also the main reason for the improvement in device performance. Meanwhile, the bias voltage value corresponding to the peak capacitance decreases, indicating that the hole injection is advanced and the carriers can recombine under a lower bias voltage condition.^[^ ^38^ ^]^

To deeply investigate the injection process of holes and electrons, the J–V characteristics of single‐carrier devices were further analyzed and discussed. The structure diagram of the single carrier device is shown in Figure S11 (Supporting Information). Among them, Figure S11c (Supporting Information) shows the J–V characteristic curves of pure electron devices (EOD) and pure hole devices (HOD). The electron current density is greater than the hole current density. After treatment with bilateral Ga‐based ligands, the electron current density decreased, and both the electron leakage and hole leakage currents also decreased. Figure 4d shows the J–V characteristic curve and regional magnification of the pure hole device. After ligand treatment, when the bias voltage rises to the turn‐on voltage, the hole current density increases significantly. This can effectively improve the carrier injection balance and enhance the exciton recombination efficiency.

The influence of Ga‐based ligands on the device performance is confirmed by further analyzing the J–V characteristic curve of the QLED device (Figure 4e). Based on the exponential relationship between current density and voltage, the J–V characteristics of QLEDs devices are divided into four different regions, showing typical nonlinear characteristics. When the applied bias voltage rises to the turn‐on voltage of QLEDs device, the holes start to inject QDs EML, and the J–V curve begins to exhibit the current characteristics of trap filling (TFL).^[^ ^39^ ^]^ In this voltage region, due to the large hole injection barrier existing at the PF HTL/QD interface, the holes are usually injected into the QD layer by tunneling and thermal excitation. The insufficient hole injection leads to a low carrier recombination rate within the device, and both the device brightness and EQE remain at a relatively low level.^[^ ^40^, ^41^ ^]^ After inserting the GAS ligand at the PF HTL/QD interface, V_TFL_ decreased from 2.6 to 2.2 V, indicating that the introduction of the GAS ligand reduced the hole injection barrier, enabling holes to be effectively injected into the QDs layer at a lower voltage, and improving the transport efficiency of holes within the QDs layer under the action of the GACl ligand. This regulatory effect has also been fully verified during the operation of QLEDs devices. The L–V curves and EQE‐V curves of QLEDs devices before and after bilateral Ga‐based ligand modification are shown in Figure 4f,g. Compared with the control group, the ignition voltage of the QLEDs device drops by 0.2 V, and it has higher luminance and EQE in the low bias voltage region, after bilateral Ga‐based ligand modification.

Based on the physical model of hole injection proposed in our previous work, due to the existence of the hole injection barrier, during the operation of QLEDs devices, there are two different operating mechanisms of hole injection. The introduction of the GAS ligand layer effectively reduces the hole injection barrier, enabling large number of holes to be injected into the QDs layer in advance. Meanwhile, the VB position of QDs in the inner layer increases after GACl treatment, forming a gradient energy level, providing an additional driving force for the transport of holes within the QDs layer. The synergistic effect of bilateral ligand modification regulates the electron–hole injection balance, effectively improves the carrier recombination efficiency during the device operation process. It reduces the internal charge accumulation of the device, which avoids the degradation of the HTL functional layer. Ga‐based ligand modification not only significantly improves the EQE of QLEDs devices, but also greatly extends the device lifetime, providing a new interface engineering strategy for the development of high‐performance blue QLEDs devices.

## 3 Conclusion

In summary, we propose an in situ dual interfaces ligand modification strategy. At the ETL/QDs interface, the introduction of GACl ligands enables synergistic defect passivation by the ─NH_2_ functional group and halide ions, reducing leakage electron current while suppressing electron injection and improving hole transport efficiency within the QDs layer. Meanwhile, at the HTL/QDs interface, modification with the GAS ligand reduces the hole injection barrier while passivating QD surface defects, thereby enhancing hole injection efficiency in the low‐voltage region and mitigating interfacial charge accumulation. The dual‐interfaces modified blue QLEDs exhibit a significant improvement in EQE from an initial 16.5% to 24.3%, along with high brightness (44100 cd m^−^^2^), high current efficiency (21.36 cd A^−1^), and remarkable device stability. The operational lifetime (T_50_@100 cd m^−^^2^) reaches 6600 h, representing a sevenfold enhancement. This innovative strategy provides a promising pathway for achieving high‐performance blue QLEDs.

## 4 Experimental Section

### Materials

Guanidinium chloride (GACl, 99%), Guanidine sulfamate (GAS, 90%) were purchased from Adamas Reagent Co., Ltd. Zinc acetate dihydrate (Zn(Ac)_2_•2H_2_O, 99.99%), Magnesium acetate tetrahydrate (Mg(Ac)_2_•4H_2_O, 99.99%), Dimethyl sulfoxide (DMSO, 99.7%) and Tetramethylammonium hydroxide pentahydrate (TMAH, 97%) were purchased from Sigma–Aldrich Co., Ltd. Poly((9,9‐dioctylfluorenyl‐2,7‐diyl)‐alt‐(9‐(2‐ethylhexyl)‐carbazole‐3,6‐diyl)) (PF8Cz) was purchased from Dongguan Fuan Optoelectronics Technology Co., Ltd. Poly(3,4‐ethylenedioxythiophene):poly(styrenesulfonate) (PEDOT: PSS, Clevios PVP AI4083) was purchased from Kurt J. Leske Co., Ltd. The CdZnSe/CdZnS/ZnS core‐shell QDs were purchased from Pujiafu Nanotech Co., Ltd. All chemical reagents were analytical grade and used without further purification.

### Fabrication of QLEDs

The patterned ITO substrates were cleaned by a sequential sonication treatment in detergent, DI water, acetone, and isopropanol (IPA) for 20 min, respectively. After drying, the ITO substrates were exposed to UV‐ozone for 30 min. PEDOT: PSS solution was spin‐coated on the ITO glass at 5000 rpm for 20 s, followed by baking at 150 °C for 15 min in air. All samples were transferred to a glove box full of N2. Then, PF8Cz dissolved in chlorobenzene as HTL was spin‐coated over the PEDOT: PSS layer at 3000 rpm for 20 s, followed by annealing at 150 °C for 30 min. The core‐shell QDs emitting layer over the PF8Cz layer was prepared as follows.

First, the ligand solution of GAS (5 mg mL^−1^) was spin‐coated at 4000 rpm for 20 s on the PF layer to form the bottom ligand layer. The QDs in octane (18 mg mL^−1^) were spin‐coated at 3000 rpm for 20 s. Guanidine‐based ligand layers were formed on the QDs film by spin‐casting guanidine‐based ligand solutions diluted in ethanol (3 mg mL^−1^) with a spin‐rate of 4000 rpm for 30 s. To remove weakly bound guanidine‐based ligands, washing steps with neat ethanol were carried out by spin‐coating, and the film was baked at 80 °C for 30 min. The colloidal solution of ZMO nanoparticles was spin‐coated at 2500 rpm for 20 s and annealed at 80 °C for 30 min. After the spin‐coated process completed, Al cathode (100 nm) was deposited by a thermal evaporator under vacuum (< 5 × 10^−6^ torr). The UV adhesive (NOA61) used in the experiment was purchased from Norland Co., Ltd. A small amount of UV adhesive was dropped on the top of the QLED device, covered with the cover glass, and cured for 2 min under the UV lamp. The active area of the devices was 0.04 cm^−2^ determined by a pattern metal mask.

### Characterization and Measurements

Film characterization: the FTIR spectra were recorded on a Bruker INVENIOS FTIR spectrometer. Steady‐state PL spectra and the TrPL spectra were measured using a JASCO FP8500 spectrofluorometer. The TrPL decay curves were fitted using a biexponential decay function:

(1) $$f\left(t\right)=A1\;\exp\;(-\frac{t}{\tau_{1}})+A2\;\exp\;(-\frac{t}{\tau_{2}})$$

where A1 and A2 are the corresponding decay amplitudes, τ_1_ and τ_2_ were the decay time constants. Absorption spectra were measured by a UV−vis absorption spectrometer (Lambda950 PerkinElmer spectrometer). TEM and HRTEM images were recorded with a FEI Talos F200S. The UPS spectra were performed on Thermo Fisher Scientific ESCALAB 250XI at a bias of −5 V using a He‐Iα (21.22 eV) UV light source. The XPS was recorded with on Al Kα X‐ray photons (hν = 1486.6 eV) using an AXIS SUPRA+ System (Shimadzu Inc.). The surface morphologies of the QDs films and c‐AFM images were obtained from Atomic Force Microscope (AFM, Dimension Icon, Bruker). Transient absorption spectroscopy measurements were tested on Femtosecond transient absorption spectrometer and Femtosecond laser amplifier system (Helios‐EOS FIRE, Coherent/Astrella‐F‐1K). The J–V–L characteristics, the EQE and current efficiency as a function of luminance, and EL spectra of the QLEDs were carried out on a characterization system comprising a Keithley 2400 voltmeter together and a Photo Research 735 (PR‐735) spectrometer under ambient conditions. Operational stability of the QLEDs under a constant current was defined by a QLED life test system of Newport Keithley N6705B.

## Conflict of Interest

The authors declare no conflict of interest.

## Supporting information



Supporting Information

## Data Availability

The data that support the findings of this study are available in the supplementary material of this article.
