# Supplementary material for: Regulation on Dual Interfaces of QD with ETL and HTL by Guanidine‐Based Ligands Enable High‐Performance Blue Quantum Dot Light‐Emitting Diodes with 24.3% External Quantum Efficiency
Source: Adv Sci (Weinh). 2025 Sep 25;12(47):e12478. doi: 10.1002/advs.202512478 (PMC12713087; doi:10.1002/advs.202512478)
Supplement: Supplementary file 1 — Supporting Information [file ADVS-12-e12478-s001.docx]

**Regulation on Dual Interfaces of QD with ETL and HTL by Guanidine-based Ligands Enable High-performance Blue Quantum Dot Light-emitting Diodes with 24.3% External Quantum Efficiency**

*Yanfang Ren, Yunqi Wang, Yan Fang, Xiaohong Jiang, Ke Cheng*, Zuliang Du**

*National & Local Joint Engineering Research Center for High-efficiency Display and Lighting Technology,*

*Key Lab for Special Functional Materials of Ministry of Education, and School of Nanoscience and Materials Engineering, Henan University, Kaifeng 475004, China Henan University, Kaifeng 475004, China*

**Corresponding author:*

*Prof. Zuliang Du, E-mail:* [*zld@henu.edu.cn*](mailto:zld@henu.edu.cn)

*Prof. Ke Cheng, E-mail:* *[ck@henu.edu.cn](mailto:ck@henu.edu.cn)*


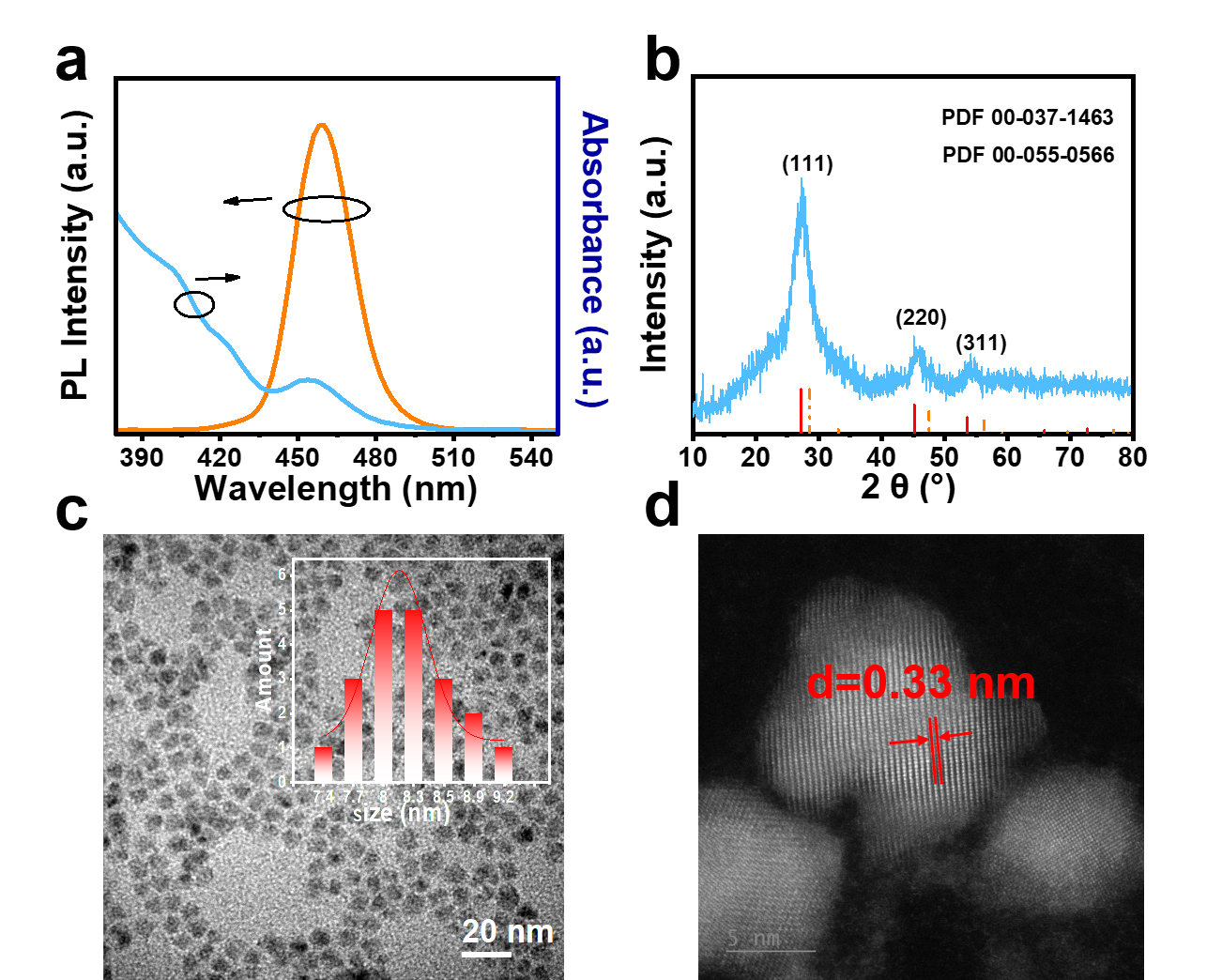


**Figure S1.** Characterization of CdZnSe/CdZnS/ZnS QDs. a) Absorption and photoluminescence spectra. b) XRD patterns, together with the reference zinc blende structure of ZnSe (X-ray diffraction pattern PDF 00-037-1463) and ZnS (PDF 00-005-0566). c) TEM images, the inset was the particle size distribution of QDs. d) HRTEM image.


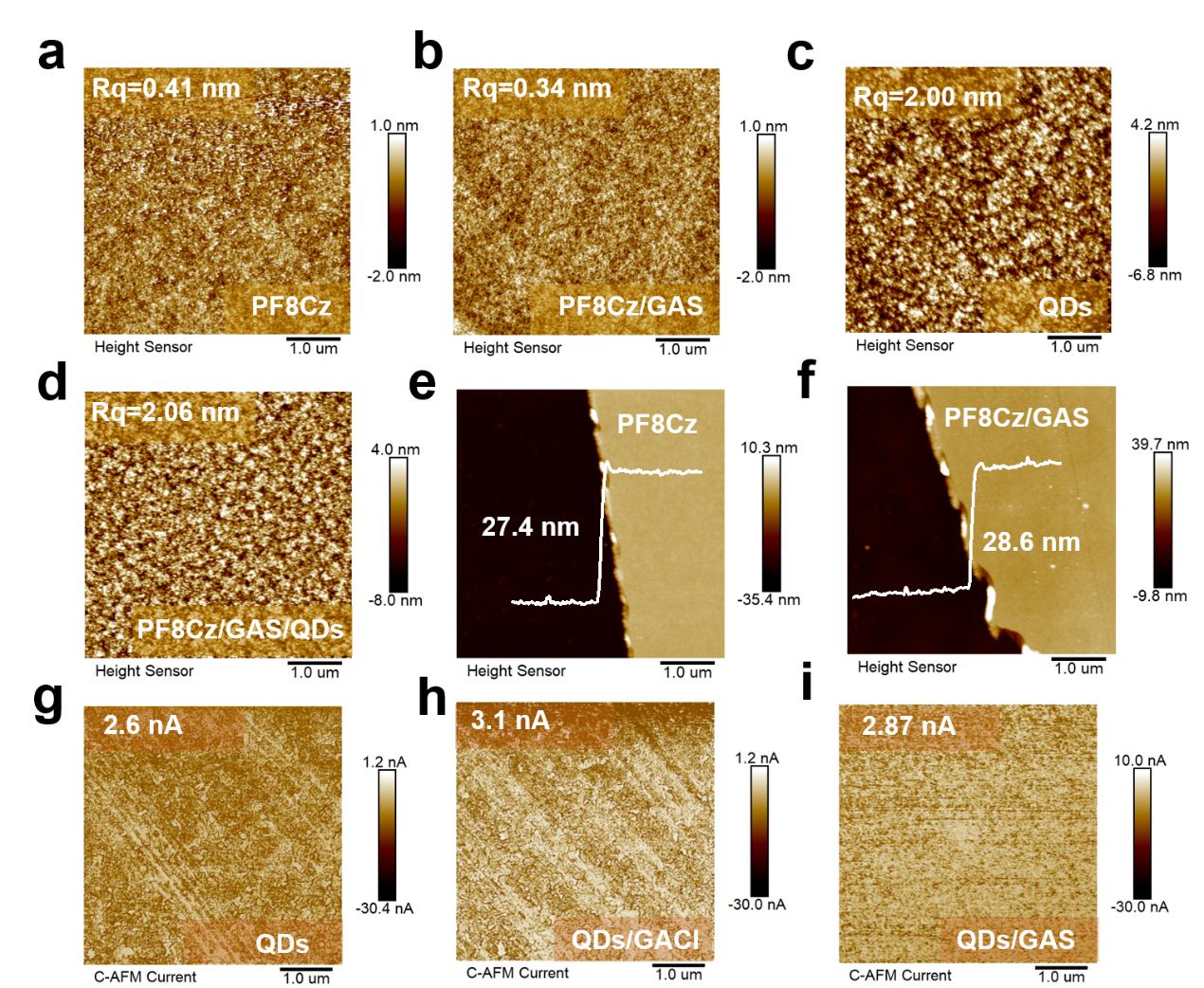


**Figure S2.** AFM images, a) PF8Cz film, b) PF8Cz/GAS composite film, c) PF8Cz/GAS/QDs, d) QDs film, e) PF8Cz film thickness, f) PF8Cz/GAS composite film thickness. C-AFM images of g) QDs film, h) QDs/GACl film, i) QDs/GAS film.


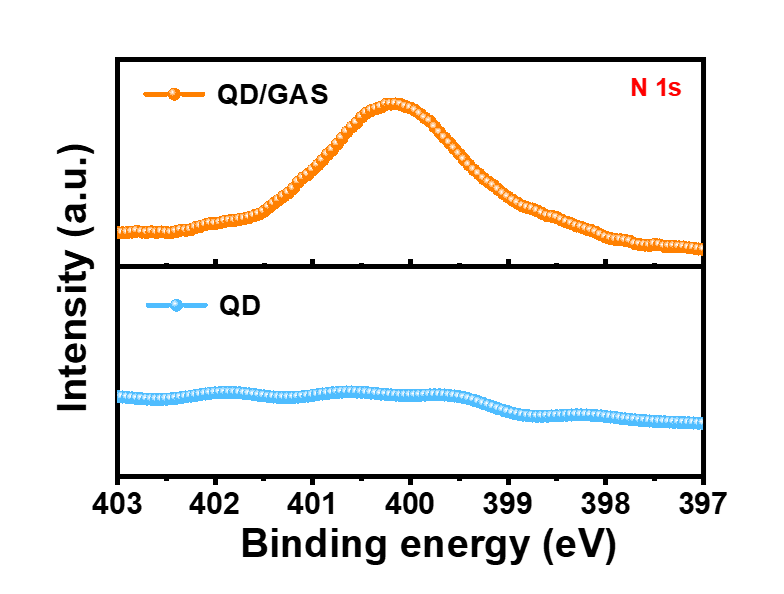


**Figure S3.** High-resolution XPS spectra of elements N 1s.


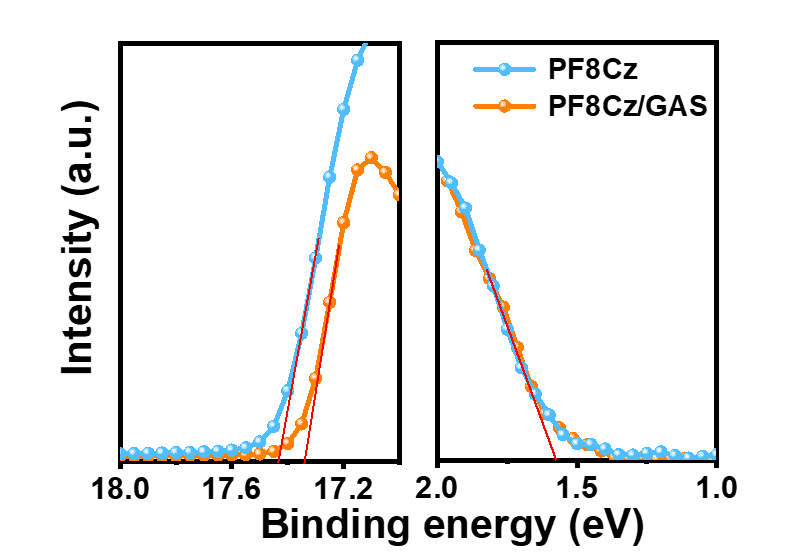


**Figure S4.** UPS spectra of PF8Cz film and PF8Cz/GAS composite film.


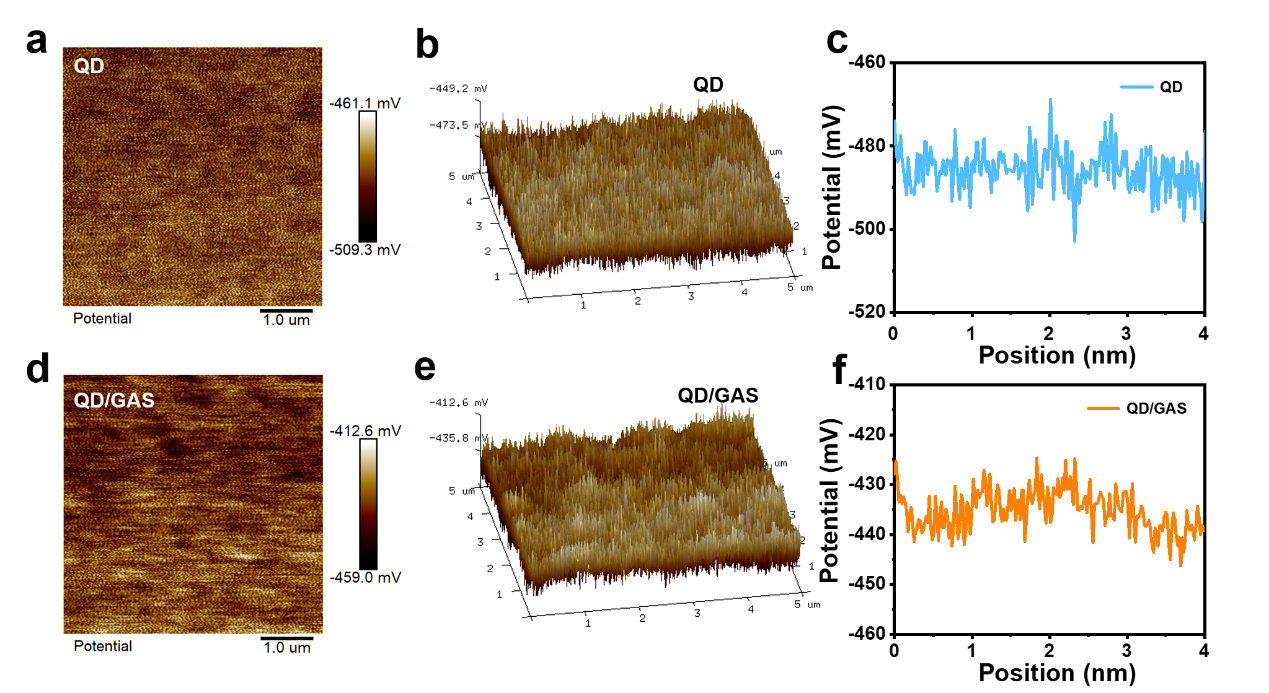


**Figure S5.** Surface potential measured by scanning Kelvin probe microscopy (SKPM). a) Surface potential images, b) 3D surface potential images and c) surface potential distribution curve of QDs film. d) Surface potential images, e) 3D surface potential images and f) surface potential distribution curve of QDs/GAS film.


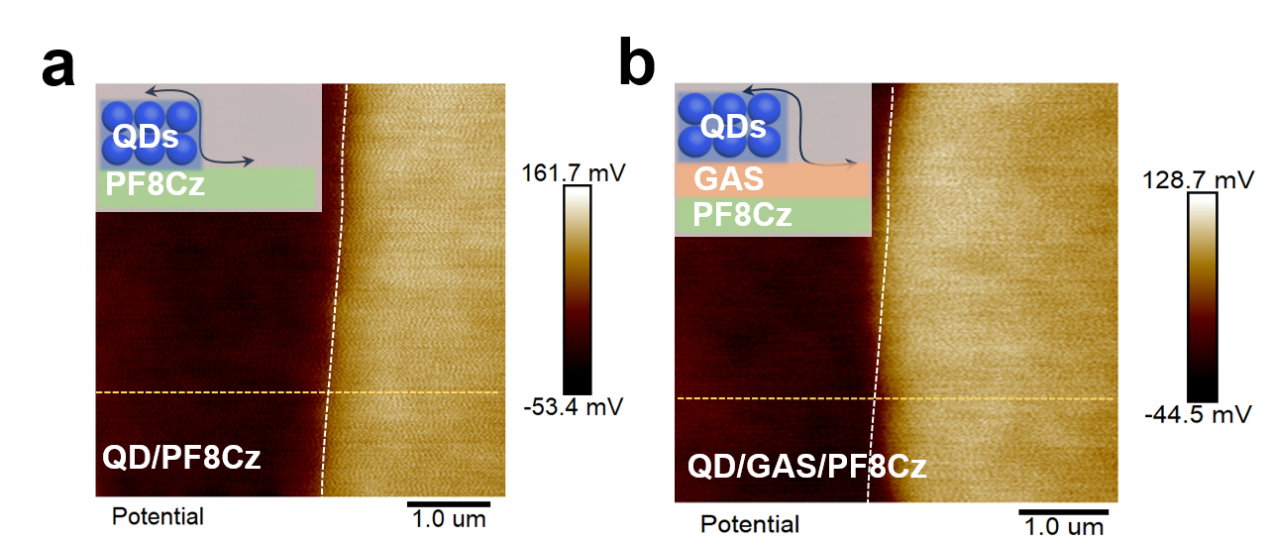


**Figure S6.** Surface potential images of a) QDs and b) QD/GAS films surface partially covered with PF8Cz HTL, respectively.


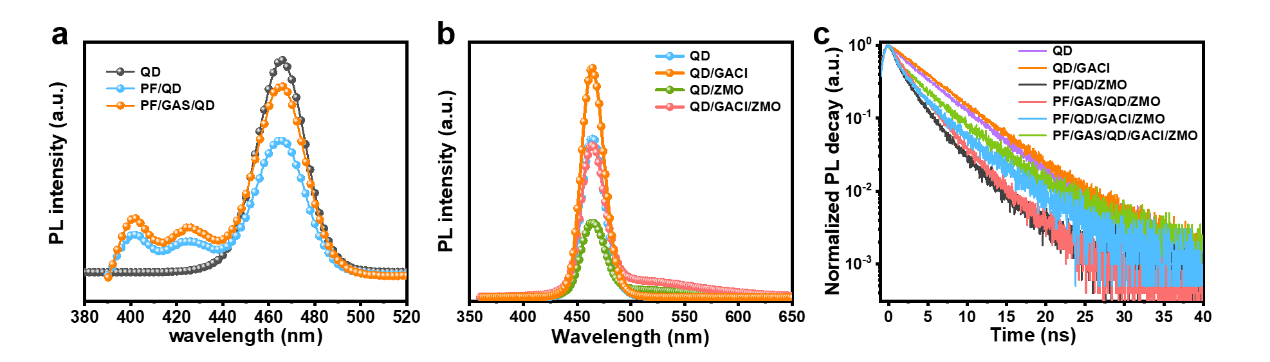


**Figure S7.** Optical properties of composite film. a) PL curves of QD and QD/HTL. b) PL curves of QD and QD/ETL. c) TrPL curves of QD, QD/HTL and HTL/QD/ETL.

**Table S1**. Summarized components of the fluorescence lifetimes of different samples.

| samples | τ (ns) |
| --- | --- |
| QDs | 5.0 |
| QDs/GACl | 5.3 |
| PF/QDs/ZMO | 3.0 |
| PF/GAS/QDs/ZMO | 3.2 |
| PF/QDs/GACl/ZMO | 3.9 |
| PF/GAS/QDs/GACl/ZMO | 4.6 |


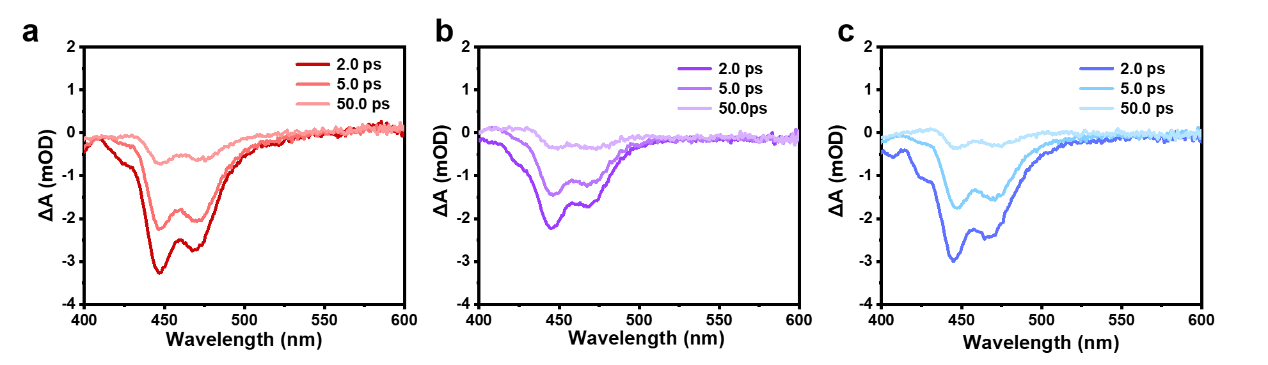


**Figure S8.** Photoexcited carrier dynamics analysis. (a)The TA spectra of QD film. (b) The TA spectra of PF/QD/ZMO composite film. (c) The TA spectra of PF/GAS/QD/GACl/ZMO.


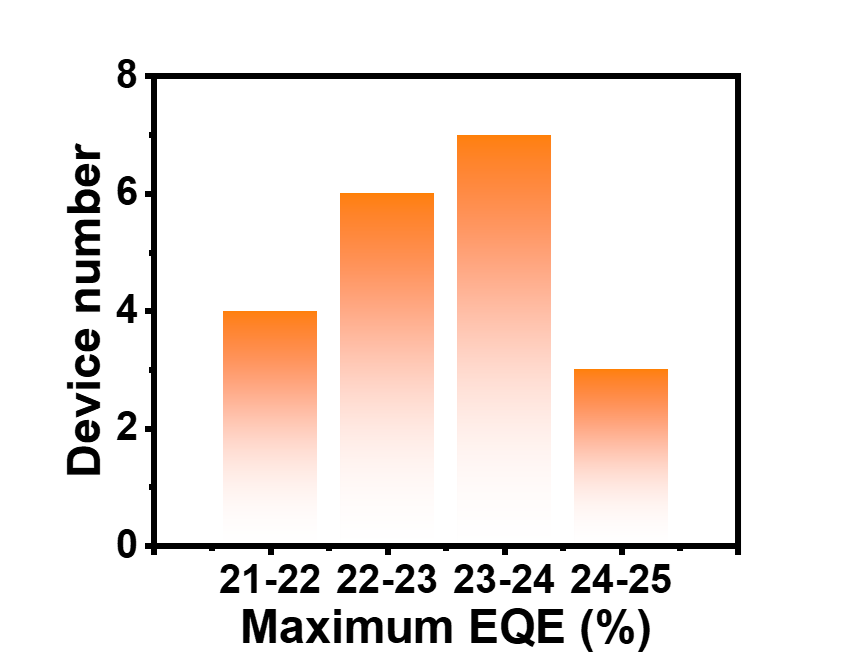


**Figure S9.** Statistical graph of the maximum EQE.

**Table S2.** Summarized Luminance, EQE, and current efficiency of QLEDs.

|  | L (cd/m^2^) | EQE (%) | η_A_ (cd/A) |
| --- | --- | --- | --- |
| Control | 27300 | 16.56 | 13.15 |
| QDs/GACl/ZMO | 35020 | 22.04 | 17.96 |
| Asymmetric GA-ligand | 44100 | 24.27 | 21.36 |


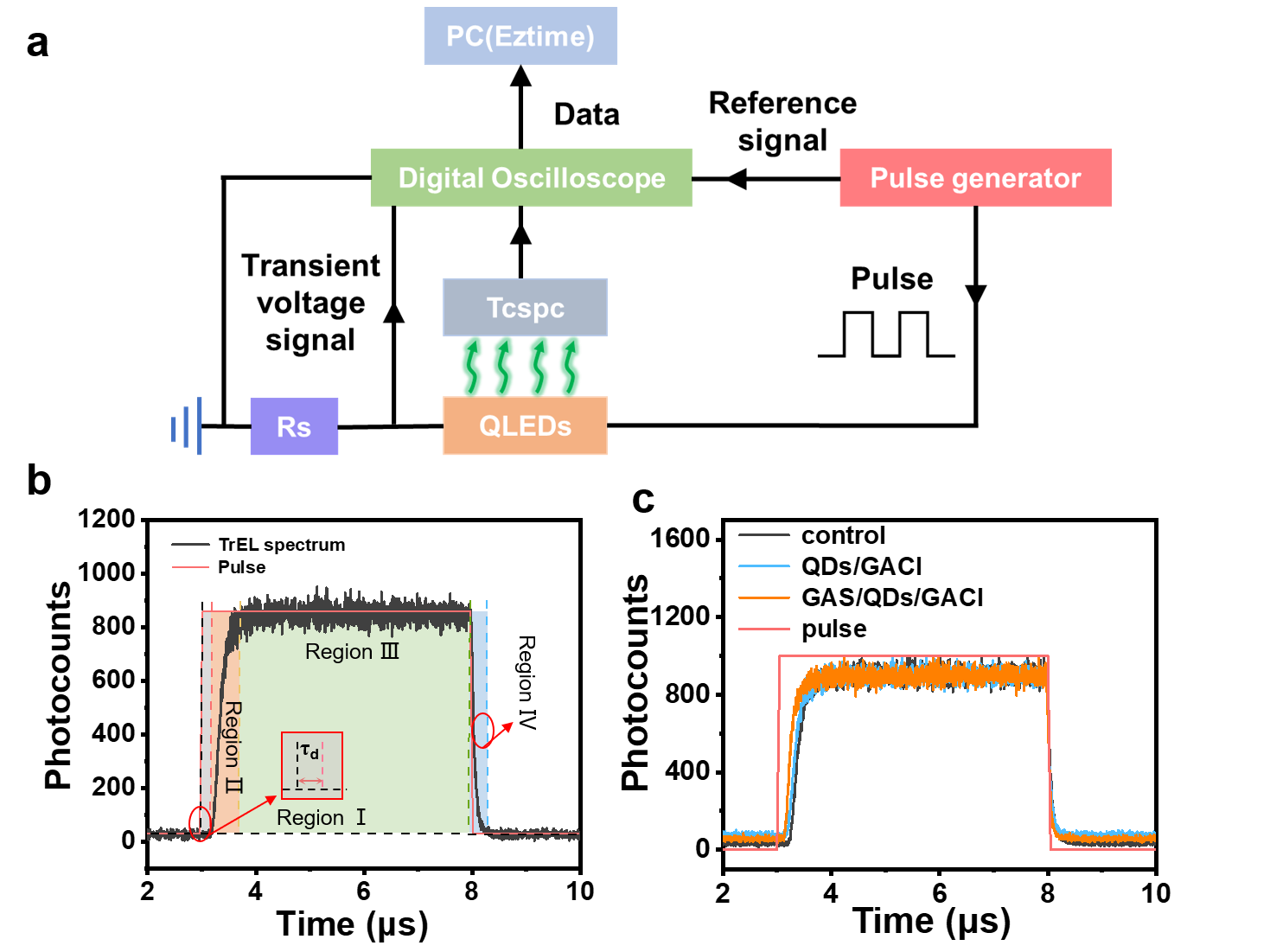


**Figure S10.** a) Conventional TrEL spectrum of QLEDs. b) The TrEL spectrums of different devices (@ 2.8 V).


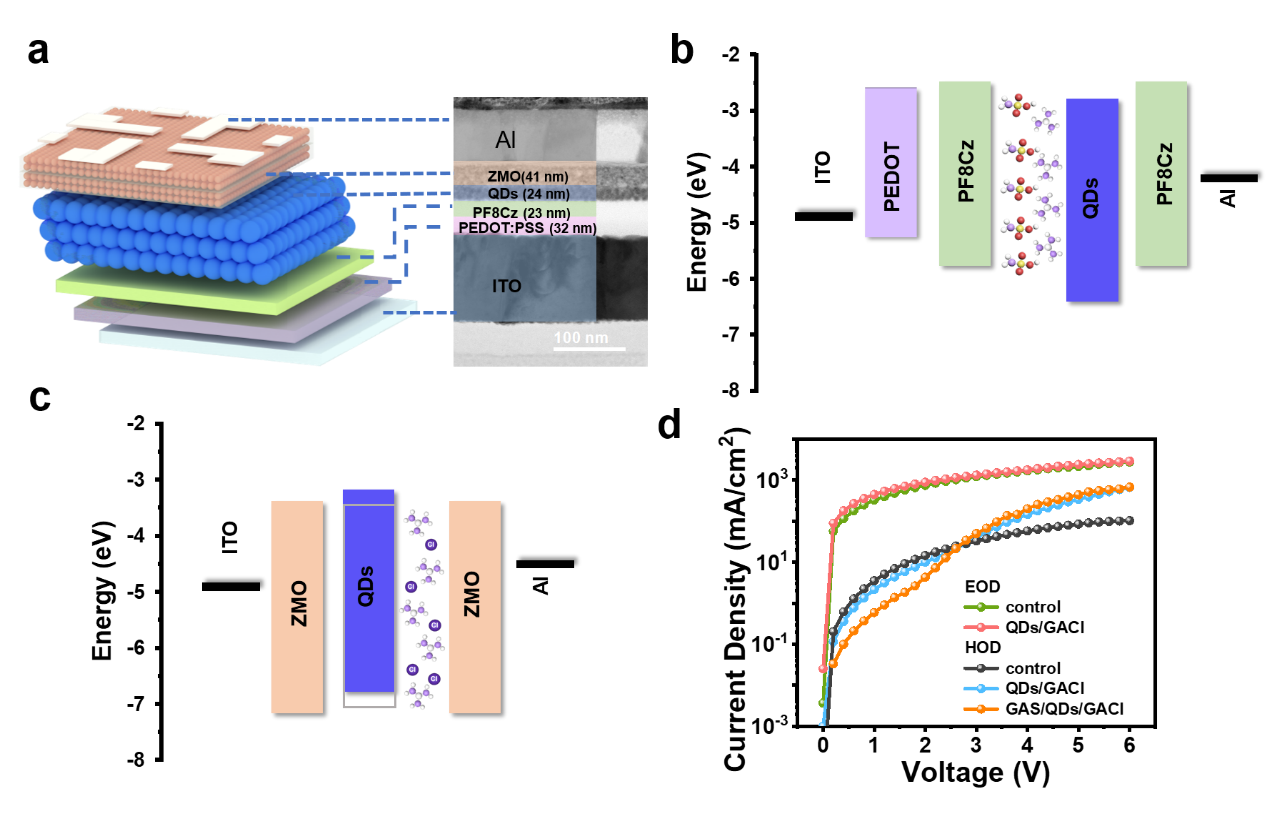


**Figure S11.** a) Device structure diagram and cross-sectional TEM image of blue QLED, ITO/PEDOT:PSS (32 nm) /PF8Cz (23 nm)/ QDs (24 nm) /ZMO (41nm) /Al. Device architecture of b) electron-only devices and c) hole-only devices. d) Current density-voltage characteristics of electron-only devices and hole-only devices.
